# Investigating the clinical advantages of a robotic linac equipped with a multileaf collimator in the treatment of brain and prostate cancer patients

**DOI:** 10.1120/jacmp.v16i5.5502

**Published:** 2015-09-08

**Authors:** Christopher M. McGuinness, Alexander R. Gottschalk, Etienne Lessard, Jean L. Nakamura, Dilini Pinnaduwage, Jean Pouliot, Colin Sims, Martina Descovich

**Affiliations:** ^1^ Department of Radiation Oncology University of California at San Francisco San Francisco CA; ^2^ Accuray Inc. Sunnyvale CA USA

**Keywords:** CyberKnife M6, CyberKnife, SBRT, SRS, micro‐MLC, InCise MLC

## Abstract

The purpose of this study was to evaluate the performance of a commercially available CyberKnife system with a multileaf collimator (CK‐MLC) for stereotactic body radiotherapy (SBRT) and standard fractionated intensity‐modulated radiotherapy (IMRT) applications. Ten prostate and ten intracranial cases were planned for the CK‐MLC. Half of these cases were compared with clinically approved SBRT plans generated for the CyberKnife with circular collimators, and the other half were compared with clinically approved standard fractionated IMRT plans generated for conventional linacs. The plans were compared on target coverage, conformity, homogeneity, dose to organs at risk (OAR), low dose to the surrounding tissue, total monitor units (MU), and treatment time. CK‐MLC plans generated for the SBRT cases achieved more homogeneous dose to the target than the CK plans with the circular collimators, for equivalent coverage, conformity, and dose to OARs. Total monitor units were reduced by 40% to 70% and treatment time was reduced by half. The CK‐MLC plans generated for the standard fractionated cases achieved prescription isodose lines between 86% and 93%, which was 2%–3% below the plans generated for conventional linacs. Compared to standard IMRT plans, the total MU were up to three times greater for the prostate (whole pelvis) plans and up to 1.4 times greater for the intracranial plans. Average treatment time was 25 min for the whole pelvis plans and 19 min for the intracranial cases. The CK‐MLC system provides significant improvements in treatment time and target homogeneity compared to the CK system with circular collimators, while maintaining high conformity and dose sparing to critical organs. Standard fractionated plans for large target volumes $\left(>100\;{\text{cm}}^{3}\right)$ were generated that achieved high prescription isodose levels. The CK‐MLC system provides more efficient SRS and SBRT treatments and, in select clinical cases, might be a potential alternative for standard fractionated treatments.

PACS numbers: 87.56.nk, 87.56.bd

PACS numbers: 87.56.nk, 87.56.bd

## I. INTRODUCTION

The CyberKnife (CK) Robotic Radiosurgery System (Accuray Inc., Sunnyvale, CA) is a medical device designed for stereotactic radiosurgery (SRS) and stereotactic body radiotherapy (SBRT) treatments. It enables the delivery of radiation from multiple beams with stereotactic precision

[a] Corresponding author: Christopher McGuinness, Department of Radiation Oncology, University of California San Francisco, 1600 Divisadero, Suite H1031, San Francisco, CA 94143-1708, USA; phone: (415) 353 9848; fax: (415) 353 9883; email: mcguinnessc@radonc.ucsf.edu provided by image guidance. Throughout the treatment, target localization is achieved by automatic registration of two orthogonal live X-ray images with a library of digitally reconstructed radiographs generated from the patient's planning CT. Image registration is based on the location of gold markers implanted in soft tissue such as prostate, or based on anatomical bony landmarks such as the skull or vertebral bodies, or based on the center of mass of lung lesions directly.[1,2,3] For tumors that move with respiration (e.g., lung, liver, pancreas), the CyberKnife correlates this image registration with continuous reading of external optical markers on the patient's chest to create a prediction model. This prediction model is continuously updated during treatment delivery with additional images so as to gradually adapt to changing respiratory pattern. This allows the robotic arm to move the beam in synchrony with the tumor as the patient breaths normally.[4] Targeting accuracy is within 1 mm for both static targets such as cranial or spinal tumors,[5,6,7] as well as dynamic targets such as lung tumors.[7,8] High targeting accuracy allows smaller planning target volume (PTV) margins. For example, Xie et al.[9] conclude a 2 mm margin for prostate cases with fiducial tracking is sufficient when imaged every 30 s, and Murphy[10] reports a reduction in margin needed for cranial and spinal radiosurgery from 4.5 mm to 1.6 mm when using dynamic skull tracking instead of static alignment.

The delivery system consists of a 6 MV linear accelerator mounted on a robotic arm that is able to deliver radiation from hundreds of robot positions spaced uniformly around the target. The beam size is controlled through either a fixed collimator system or a variable aperture Iris collimator (Accuray Inc.), both with the choice of 12 circular beams of diameters ranging from 5 mm to 60 mm[11,12] or via the newly introduced MLC system. In a typical CK plan, hundreds of nonisocentric and noncoplanar circular radiation beams are pointed to the edge of the target, creating a highly conformal dose distribution with sharp dose drop-off at its periphery and low dose to adjacent organs at risk (OARs).[13] These characteristics make CyberKnife ideal for treatments that require high spatial accuracy and high conformity, such as SRS and SBRT treatments.

However, the use of a large number of circular radiation fields to cover the target has four main limitations: 1) the radiation delivery is inefficient and a large number of monitor units (MU) are required to deliver the desired dose; 2) the treatment times are long as, for safety reasons, the robot travels from one position to the next at a speed of 60–80 mm/s, 1/30th of its rated top speed. Treatment time including setup, imaging, beam on, and robot motion is usually between 30 min and 1 hr; 3) dose to the target is inhomogeneous, with prescription isodose lines typically ranging between 50% and 85% of the maximum dose (while this is desirable for most SBRT plans, it limits the application of CK to other treatments); 4) larger target volumes are not treated on CyberKnife due to the longer treatment delivery time associated with the larger number of beams required and the difficulty in achieving satisfactory target coverage by targeting small pencil beams to the edge of the target (dose painting).

The duration of a treatment can be reduced by the application of time-reduction techniques aimed at reducing the number of beams and the total MUs.[7,14] In addition, the Iris collimator enables the use of multiple beam apertures in a single path traversal. Although these techniques are successful in reducing the fraction duration by tens of minutes, total treatment times remain long for complex clinical cases due to the inherent limitations of using circular radiation fields. A number of feasibility studies have shown that the addition of a MLC to the CyberKnife system would reduce treatment time and total monitor units, and improve plan quality.[15,16,17] A MLC reduces the number of beams by eliminating the need of dose painting with multiple circular radiation fields. The addition of a MLC to the CK system could reduce the MU and treatment time, increase target dose homogeneity, and allow the treatment of larger targets.

Previous studies have generated plans for theoretical CK models equipped with MLC, using either in-house developed optimization software[17] or treatment planning system for linac-based SBRT (Eclipse) combined with manual beam angle selection.[14] Today, a new CK system equipped with three interchangeable collimator systems (fixed, Iris, and MLC) is available for clinical use. This device has the potential to improve efficiency of SRS and SBRT treatments and to extend the advantages of noncoplanar treatment[18] and real-time tracking[4] to patients treated with standard fractionation.

The CK InCise multileaf collimator system (Accuray Inc.) consists of 41 leaf pairs, with a thickness of 9 cm and a width of 2.5 mm at 80 cm SAD. The maximum field size is 12 cm in the direction of leaf motion and 10 cm in the vertical direction at 80 cm SAD. The leaf motion allows 100% over-travel and interdigitation. Intraleaf, interleaf, and leaf tip leakage are all specified to be less than 0.5% maximum (0.3% average).[19] Beam parameters were measured by the vendor on a prototype machine and used to commission the treatment planning system used in this study. For comparison, the clinically approved IMRT plans were generated for the Siemens Artiste with the 160 MLC consisting of 80 leaf pairs of 5 mm width and a specified maximum leakage of less than 1.5%.[20]

The goal of this study is to provide an initial demonstration of the clinical capabilities of the CK-MLC system in the treatment of brain and prostate cancer patients. Figure 1 presents the organization of the types of cases included in this study. To the authors' knowledge, this is the first study comparing treatment plans generated for a commercially available CK-MLC system to: 1) CK plans generated with circular collimators (fixed or Iris), and 2) standard fractionated intensity-modulated radiation therapy (IMRT) plans generated for a standard linac. We chose to study brain and prostate SBRT cases because they are some of the most common sites currently treated with CyberKnife at our institution in addition to lung cases. Currently, the finite size pencil beam (FSPB) dose calculation algorithm available for the CK-MLC system does not account for changes in the lateral electron scattering fluence due to tissue inhomogeneities.[21] This might result in large dose calculation errors in regions with density interfaces. While lung tumors are a common treatment site for CK and ones that could benefit from CK-MLC system, we did not include any lung cases in this comparison because of errors introduced by tissue inhomogeneity. The target volume for brain and prostate cases is surrounded by homogeneous tissue and dose calculation is not affected by the presence of tissue inhomogeneities. We chose intracranial and whole pelvis IMRT cases to explore the benefit of highly conformal dose distribution for targets in close proximity to OARs, such as the brain stem and optical chiasm for the intracranial cases and the bladder and rectum for the whole pelvis cases. The whole pelvis plans, in particular, were chosen to investigate the capabilities of treating large target volumes. These cases comprise a diverse set of plans with a wide range of target volumes, proximity to OARs, and clinical objectives, providing a broad scope of the capabilities and limitations of the CK-MLC system.

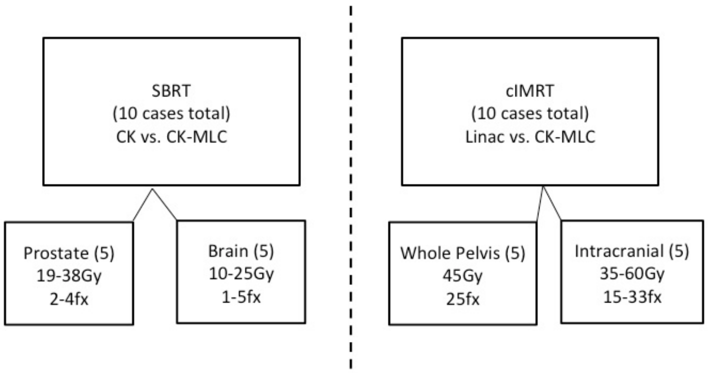

Fig. 1. This study considers 10 SBRT plans and 10 conventional IMRT plans. Five prostate and five brain cases where chosen for the SBRT comparison between CK with circular collimators (fixed and Iris) and CK with the MLC collimator. Five whole pelvis and five intracranial cases were chosen for the standard fractionated IMRT plans, comparing the CK-MLC to plans generated for conventional linacs.

## II. MATERIALS AND METHODS

### A. Patients

In this study, we included a total of 20 patients (10 brain and 10 prostate) treated at our institution. Half of these cases were SBRT plans, previously treated on CK, while the remaining half were IMRT plans generated in Pinnacle (Philips Healthcare, Andover, MA) for a Siemens linear accelerator (Siemens AG, Erlangen, Germany) with standard fractionation of 1.8 to 2 Gy per fraction. We selected a variety of clinical cases, to test the capability of the CK-MLC under different conditions.

The details for each case are summarized in Table 1. Five were prostate cases with planning target volumes (PTV) ranging from 33 to 85 $cm^3$ treated on CK with SBRT. Five were brain lesions with PTVs ranging between 7 and 70 $cm^3$ treated on CK with SBRT. Case 9 consisted of a 69 $cm^3$ planning target volume, with a simultaneous integrated boost to a 50 $cm^3$ gross tumor volume (GTV). Five cases included the prostate plus lymph nodes and seminal vesicles originally planned in Pinnacle for IMRT. Four of the five cases had target volumes between 425 and 500 $cm^3$, while Case 15 had a larger target volume of 760 $cm^3$ due to more extensive nodal volume included in the PTV. The last five cases were intracranial tumors with PTVs ranging between 45 and 220 $cm^3$ planned in Pinnacle for IMRT. All of the Pinnacle plans were step-and-shoot plans with seven to nine fields.

Table 1. Summary of cases included in this study. All cases presented here had a single PTV, except Case 7 which had three separate PTVs, Case 18 which had a boost volume within the larger PTV. Case 9 was a large brain metastasis which had a higher prescription to the CTV and lower prescription to the PTV. The diameter presented here is the maximum of the sup/inf, ant/post, and left/right dimensions.

| *Case #* | *Site* | *Modality* | *Diameter (cm)* | *PTV ($cm^3$)* | *Rx (Gy)* | *Fractions* |
|---|---|---|---|---|---|---|
| 1 | Prostate | SBRT | 5.5 | 56.8 | 38 | 4 |
| 2 | Prostate | SBRT | 5.9 | 84.48 | 38 | 4 |
| 3 | Prostate | SBRT | 5.0 | 42.02 | 38 | 4 |
| 4 | Prostate | SBRT | 4.3 | 33.27 | 19 | 2 |
| 5 | Prostate | SBRT | 5.9 | 78 | 19 | 2 |
| 6 | Brain mets | SBRT | 4.2 | 36.15 | 25 | 5 |
| 7 | Brain mets | SBRT | 4, 2, 1.8 | 29.5 | 24 | 3 |
| 8 | Brain mets | SBRT | 2.2 | 7.37 | 18 | 1 |
| 9 | Brain mets | SBRT | 4.7 | 49.7 / 68.8 | 20 / 15 | 5 |
| 10 | Brain mets | SBRT | 3.5 | 25.18 | 10 | 1 |
| 11 | Whole Pelvis | IMRT | 14.1 | 463.82 | 45 | 25 |
| 12 | Whole Pelvis | IMRT | 13.9 | 426.25 | 45 | 25 |
| 13 | Whole Pelvis | IMRT | 13.2 | 428.43 | 45 | 25 |
| 14 | Whole Pelvis | IMRT | 13.7 | 490.24 | 45 | 25 |
| 15 | Whole Pelvis | IMRT | 18.3 | 760.23 | 45 | 25 |
| 16 | Intracranial | IMRT | 8.3 | 218.61 | 59.4 | 33 |
| 17 | Intracranial | IMRT | 6.4 | 52.36 | 50.4 | 28 |
| 18 | Intracranial | IMRT | 10 | 80.5 / 176.8 | 40.5 / 35 | 15 |
| 19 | Intracranial | IMRT | 5.6 | 46.92 | 60 | 30 |
| 20 | Intracranial | IMRT | 6.6 | 106.66 | 50.4 | 28 |

### B. Planning strategy and evaluation

The CK-MLC plans were generated using a treatment planning system provided by Accuray (MultiPlan 5.0; Accuray Inc, Sunnyvale, CA). The beam model used in this system is based on beam parameters measured from a prototype machine at Accuray. Plans were optimized using the sequential optimization method.[22,23] We limited the number of starting robot positions to 60 for most cases.

The tracking capability of CK allows for target margins reduction compared to conventional linacs without stereotactic imaging and motion tracking. However, in our study, GTV/CTV to PTV margins were kept the same between the two plans in order to focus on the difference between the treatment machines and planning software. PTV margins of 2 mm were used for the SBRT cases, and 5 mm for the standard fractionated plans.

The dose-volume constraints and objectives for the PTV and the OARs were based on dose-volume parameters in clinically approved plans. For the SBRT prostate cases, heterogeneous dose distributions are often acceptable if the urethra is spared and the hot spot is located in the lobe. Hence, tight constraints on the maximum dose to the PTV were not used in either the CK-MLC or the circular collimator plans. Maximum DVH objectives were applied to the bladder and rectum to limit the volume of either OAR receiving 75% of the prescription dose. We used the method described in Descovich et al.[24] to determine the optimal V75% for each patient anatomy. Both the circular and MLC SBRT plans used this for the maximum DVH objective as a starting point. For the SBRT brain cases, conformity was prioritized over homogeneity. Differently than for standard fractionated IMRT cases, dose heterogeneity inside the target is often desirable in SBRT cases. The minimum MU per beam were adjusted to keep at least 2 MU per beam per fraction. To justify this minimum MU cutoff, we measured the linearity of 2 MU beams with respect to 200 MU beams on our CK VSI system using the 60 mm cone at 1.5 cm depth and find agreement to be better than 1%.

Segments shapes are created by the planning system during optimization. The user has the option to specify whether to use eroded shapes, perimeter shapes, and/or random shapes, and can specify MLC leaf margin and justification (leading edge, trailing edge, or middle). Figure 2 shows examples of each of these. Eroded shapes are some fraction of the entire PTV from a beams eye view, perimeter shapes are a narrow fields around the perimeter of the PTV, and random shapes are random, as the name implies, within the PTV determined by the planning system. A new feature in this version of the planning system is to allow beams that exit, but do not enter, through OARs. We used all of these shapes for our plans.

We evaluate plan quality based on target coverage, homogeneity, conformity, dose to critical organs, low dose to surrounding tissue (R50%), and plan efficiency (total MU and treatment time). We enforced target coverage to be greater than or equal to 95% for all the SBRT plans. Homogeneity was quantified by the prescription isodose, given as a percentage of the maximum dose ($D_{max}$). The new conformity index is an extension of the standard conformity index that reflects how tightly the prescription dose overlaps the target volume. We use the same definition as MultiPlan, described by Nakamura:[25]

$$nCI = PTV * PIV / TIV^2 \quad (1)$$

where *TIV* is the tumor isodose volume and *PIV* is the prescription isodose volume. This definition is the inverse of that used by Paddick[26] and van't Riet et al.[27] Low dose to surrounding

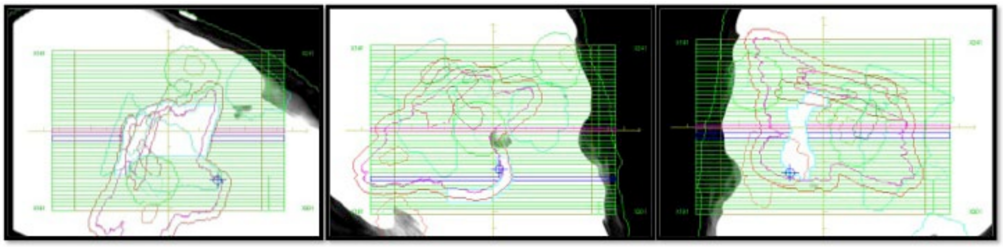

Fig. 2. The planning system automatically derives MLC shapes during the optimization process. The user has the option to use eroded, perimeter, and/or random shapes. Examples of each of these are shown here with an eroded shape on the left, a perimeter shape in the center, and a random shape on the right.

tissue was quantified using R50%, the ratio of the volume receiving 50% of the prescribed dose to the PTV:

$$R50\% = V50\%/PTV \quad (2)$$

The estimated treatment time is based on measurements performed by Accuray on a prototype machine and takes into account the number of robot positions, the robot speed traveling from one position to another (60–80 mm/s), the number of beam angles and MLC segments, the time to adjust the beam angles and to position the leaves which are done in parallel (2 s), the number of monitor units, and the dose rate (1000 MU/min). The imaging acquisitions are also taken into account based on imaging frequency. For comparison, the dose rate for the conventional linac was 300 MU/min. Treatment times presented here do not include setup time for either modality.

Dose to the bladder, rectum, brainstem, and chiasm was quantified by the generalized equivalent uniform dose (gEUD). gEUD is a concept for summarizing the entire DVH in a single metric, defined as the dose which would have the same biological effect if delivered uniformly to the entire volume. It is defined as

$$gEUD = \frac{1}{V} (\Sigma_{j=1}^{k} v_j * d_j^{1/n})^n \quad (3)$$

where $V$ is the total volume of the OAR, $v_j$ is the volume receiving dose $d_j$, and $n$ is the volume effect parameter which quantifies the degree to which the organ is serial or parallel.[28] Here we use the formalism n = 1/a, where "a" was the original volume effect parameter used by Niemierko.[29,30] An entirely serial organ is represented by n = 0 and gEUD approaches $D_{max}$. For an entirely parallel organ, n = 1 and gEUD approaches the mean dose. We use the volume effect parameters referenced in Luxton et al.[28] originally derived by others:[31,32] $n_{bladder} = 0.5$, $n_{rectum} = 0.12$, $n_{brainstem} = 0.16$, and $n_{chiasm} = 0.25$. The values presented here were derived by exporting the DVH as a table of values and computing them directly using the gEUD equation above.

Each of the metrics used to assess plan quality were averaged among the five plans in each group. The standard deviation and the p-values (two-tailed Student's $t$-test) were computed between the two comparison arms. Despite the small sample size in each group, there were statistically significant differences observed between the two arms.

## III. RESULTS

### A. SBRT cases

Results for the SBRT prostate and brain cases are summarized in Table 2. For both prostate and brain cases, the MLC plans were prescribed to high isodose levels. The nCI was statistically equivalent on average for both sites ($p = 0.67$ and $p = 0.59$, for prostate and brain, respectively). gEUD to the bladder was 25% lower on average with the MLC ($p = 0.005$). gEUD to the rectum was statistically equivalent between both modalities ($p = 0.17$). The gEUD to the optical chiasm and brainstem were lower for all MLC plans, except the dose to the chiasm in Case 9 was 0.03 Gy higher for the MLC plan, and the dose to the brainstem in Case 10 was 0.03 Gy higher for the MLC plan. Case 6, in particular, shows a large reduction in dose to the chiasm and brainstem: gEUD is reduced from 5.2 Gy to 0.8 Gy for the brain stem and 5.4 Gy to 0.16 Gy for the optical chiasm. Figure 3 shows the dose distributions for these plans. There are several beams passing through the brain stem and chiasm for the CK plan with circular collimators, which was not the case for the CK-MLC plan. MLC planning naturally minimized the low dose extension often seen with circular collimators. It should be noted that both plans respect the clinical objectives and optimization parameters. We could have removed this low dose extension using a shell or a lower MU/beam or a lower dose volume constraint. On average

Table 2. Results from five prostate (top) and five brain (bottom) SBRT cases. Values in italics are from CK plans with circular collimator and values in bold are from CK-MLC plans. The average, standard deviation, and p-values for each column are given at the bottom of the table.

| *Case #* | *Coverage (%)* | *Isodose (%)* | *nCI* | *Bladder gEUD (Gy)* | *Rectum gEUD (Gy)* | *R50%* | *Num. of Beams* | *Total MU ($10^3$)* | *Time (min)* |
|---|---|---|---|---|---|---|---|---|---|
| 1 | *94.8*\|**95.4** | *68*\|**75** | *1.12*\|**1.17** | *11.6*\|**9.2** | *19.5*\|**17.9** | *3.56*\|**3.18** | *265*\|**118** | *54.3*\|**31.3** | *47*\|**26** |
| 2 | *95.5*\|**95.1** | *66*\|**69** | *1.20*\|**1.16** | *14.1*\|**10.9** | *20.3*\|**19.8** | *3.34*\|**3.06** | *224*\|**79** | *52.2*\|**36.9** | *42*\|**23** |
| 3 | *95.2*\|**95.1** | *65*\|**67** | *1.22*\|**1.34** | *10.1*\|**8.3** | *19.5*\|**19.8** | *3.58*\|**3.53** | *170*\|**58** | *46.2*\|**26.5** | *37*\|**19** |
| 4 | *94.7*\|**96.0** | *72*\|**72** | *1.32*\|**1.27** | *5.5*\|**4.5** | *10.3*\|**10.2** | *3.95*\|**3.95** | *112*\|**50** | *22.3*\|**17.7** | *32*\|**21** |
| 5 | *94.9*\|**95.4** | *70*\|**74** | *1.28*\|**1.28** | *6.1*\|**3.3** | *9.9*\|**8.8** | *3.69*\|**3.65** | *238*\|**67** | *23.7*\|**12.1** | *44*\|**20** |
| Avg | *95.0*\|**95.4** | *68*\|**71** | *1.23*\|**1.24** | *9.5*\|**7.2** | *15.9*\|**15.3** | *3.63*\|**3.48** | *202*\|**74** | *39.7*\|**24.9** | *40*\|**22** |
| Std | *3.3*\|**0.3** | *2.9*\|**3.3** | *0.08*\|**0.08** | *3.6*\|**3.2** | *5.3*\|**5.4** | *0.22*\|**0.37** | *61*\|**27** | *15.6*\|**10.0** | *6*\|**3** |
| *p* | 0.17 | 0.048 | 0.67 | 0.005 | 0.17 | 0.13 | .003 | 0.010 | 0.001 |

| *Case #* | *Coverage (%)* | *Isodose (%)* | *nCI* | *Chiasm gEUD (Gy)* | *Brainstem gEUD (Gy)* | *R50%* | *Num. of Beams* | *Total MU ($10^3$)* | *Time (min)* |
|---|---|---|---|---|---|---|---|---|---|
| 6 | *95.5*\|**96.1** | *64*\|**82** | *1.10*\|**1.11** | *5.43*\|**0.16** | *5.20*\|**0.81** | *2.76*\|**2.67** | *197*\|**30** | *19.4*\|**5.2** | *36*\|**15** |
| 7 | *97.2*\|**95.2** | *64*\|**78** | *1.21*\|**1.21** | *1.74*\|**0.30** | *6.45*\|**6.14** | *4.39*\|**3.5** | *166*\|**45** | *39.6*\|**12.1** | *41*\|**20** |
| 8 | *94.4*\|**94.7** | *75*\|**75** | *1.10*\|**1.11** | *0*\|**0** | *1.14*\|**0.79** | *3.40*\|**2.65** | *193*\|*53* | *7.3*\|**2.8** | *34*\|**19** |
| 9 | *96.0*\|**95.3** | *80*\|**81** | *1.10*\|**1.10** | *5.77*\|**5.80** | *6.24*\|**5.75** | *4.36*\|**2.72** | *75*\|**32** | *7.1*\|**3.0** | *14*\|**11** |
| 10 | *96.8*\|**96.3** | *70*\|**73** | *1.08*\|**1.07** | *0.82*\|**0.05** | *0.28*\|**0.31** | *2.73*\|**2.39** | *172*\|**43** | *5.2*\|**2.2** | *29*\|**18** |
| Avg | *96.0*\|**95.5** | *70.6*\|**77.8** | *1.12*\|**1.12** | *4.42*\|**1.52** | *3.86*\|**2.76** | *3.53*\|**2.79** | *161*\|**41** | *15.7*\|**5.1** | *31*\|**17** |
| Std | *1.1*\|**0.7** | *7.0*\|**3.8** | *0.05*\|**0.05** | *3.81*\|**2.96** | *2.93*\|**2.92** | *0.82*\|**0.42** | *50*\|**9.5** | *14.5*\|**4.1** | *10*\|**4** |
| *p* | 0.36 | 0.12 | 0.59 | 0.085 | 0.25 | 0.05 | 0.005 | 0.085 | 0.056 |

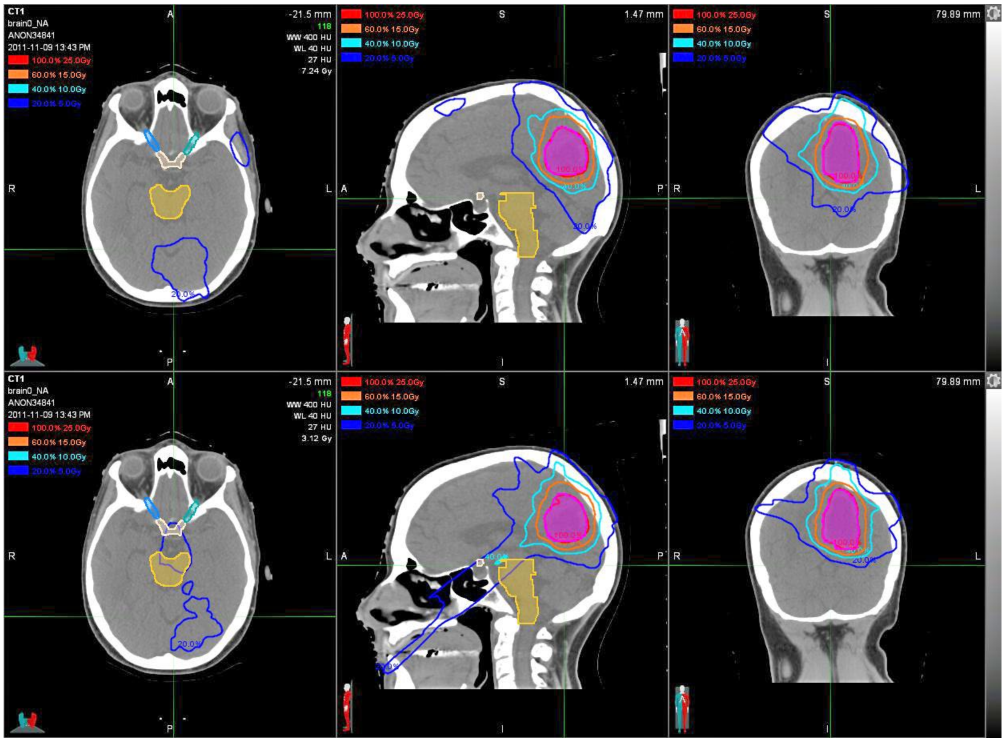

Fig. 3. CT images with isodose lines for Case 6 show a reduction in dose to the brainstem and chiasm. Several beams pass through these OARs for the CK plan with circular collimators (bottom) which was not the case for the CK-MLC plan (top).

these differences were not statistically significant ($p = 0.085$ and $p = 0.25$). R50% is lower for the MLC plans for all five brain cases ($p = 0.05$), and four of the five prostate cases ($p = 0.13$). The MLC plans used 40% fewer MUs on average for the prostate plans ($p = 0.01$) and 70% less MUs for the brain plans ($p = 0.085$). Treatment times were reduced almost in half for both sites ($p = 0.001$ and $p = 0.056$).

The DVH for all 10 prostate and brain SBRT plans were averaged and plotted in Fig. 4. Good target coverage was achieved for both modalities in both sites. For the prostate cases, the MLC plans were better able to spare the urethra and bladder and, for the brain plans, the MLC was better able to spare the brainstem and chiasm.

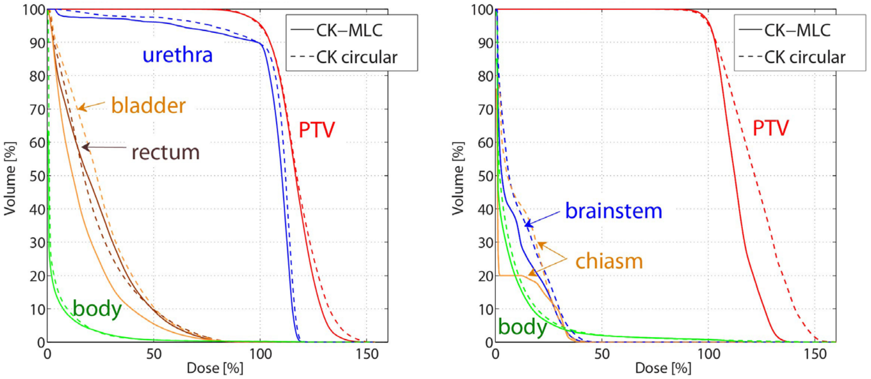

Fig. 4. Average DVHs for the five prostate (left) and five brain (right) SBRT plans. The solid lines are for the CK-MLC plans and the dashed lines are for the CK with circular collimators. The body includes the full external contour of the patient's body, defined by a lower density cutoff of 0.8 g/cm$^3$ and manually adjusted by the user when necessary. We use 0.8 g/cm$^3$ at UCSF which is slightly below that of adipose tissue (0.9 g/cm$^3$) to ensure we are including the entire body within the contour.

## B. IMRT cases

Results for the whole pelvis and intracranial IMRT plans are summarized in Table 3 and in average DVH plots in Fig. 5. Figure 6 shows the isodose lines for Case 11, with the CK-MLC plan on top and the clinically approved IMRT plan on bottom. For the whole pelvis cases, the CK-MLC plans achieved an average prescription isodose of 85.8% compared to 89.0% for clinically approved conventional linac plans ($p = 0.018$). For the intracranial cases, the CK-MLC plans achieved an average prescription isodose of 91.8% compared to 92.6% for clinically approved linac plans ($p = 0.29$). Coverage was greater than 95% for all plans, except the CK-MLC plan for Case 12 was 93.8%. For nine out of ten cases CK-MLC plans resulted in better conformity compared to conventional linac plans, though it was not statistically significant for either site ($p = 0.11$ and $p = 0.14$). The gEUD to the bladder was statistically equivalent ($p = 0.58$) and the difference was within 2 Gy for all whole pelvis plans. The gEUD to the rectum was 4 Gy larger for the CK-MLC plan for Case 14 and within 2 Gy for the remaining four cases. The differences were not statistically significant ($p = 0.8$). The difference in gEUD to the chiasm and brainstem was less than 4 Gy in all cases and statistically equivalent between the two planning systems ($p = 0.46$ and $p = 0.29$). The low dose conformity metric, R50%, was 25%–30% lower, on average, for the CK-MLC plans. This difference was statistically significant for the whole pelvis cases ($p = 0.0007$), but not for the intracranial cases ($p = 0.16$). The total number of beams and MUs for the CK-MLC whole pelvis plans was a factor of 3 greater than for the linac plans. For the intracranial cases, the total monitor units were 15,000, on average, for the CK-MLC plans compared to 11,000 for the linac plans, a factor of 1.4 larger. The estimated treatment time without setup time was 25 min, on average, for the whole pelvis CK-MLC plans, compared to 11 min, on average, for the conventional linac plans. Estimated treatment time for the intracranial cases planned for the CK-MLC were less than 20 min on average, compared to 11.5 min for the conventional linac plans.

TABLE 3. Results from five whole pelvis (top) and five intracranial (bottom) IMRT cases. Values in italics are from plans generated for a Siemens linac and values in bold are from CK-MLC plans. The average, standard deviation, and p-values for each column are given at the bottom of the table.

<table>
<tr><th>Case #</th><th>Coverage (%)</th><th>Isodose (%)</th><th>nCI</th><th>Bladder gEUD (Gy)</th><th>Rectum gEUD (Gy)</th><th>R50%</th><th>Num. of Beams</th><th>Total MU ($10^3$)</th><th>Time (min)</th></tr>
<tr><td>11</td><td>*96.3*|**95.7**</td><td>*88*|**86**</td><td>*1.35*|**1.27**</td><td>*33.7*|**35.3**</td><td>*32.2*|**33.7**</td><td>*8.12*|**5.80**</td><td>*55*|**157**</td><td>*21.0*|**72.0**</td><td>*10*|**25**</td></tr>
<tr><td>12</td><td>*95.2*|**93.8**</td><td>*89.6*|**85**</td><td>*1.52*|**1.50**</td><td>*34.6*|**33.6**</td><td>*37.3*|**33.5**</td><td>*9.58*|**6.5**</td><td>*53*|**145**</td><td>*24.0*|**78.2**</td><td>*17*|**25**</td></tr>
<tr><td>13</td><td>*96.9*|**96.6**</td><td>*90*|**88**</td><td>*1.60*|**1.53**</td><td>*31.9*|**31.3**</td><td>*33.8*|**34.2**</td><td>*9.8*|**6.43**</td><td>*57*|**173**</td><td>*22.3*|**61.7**</td><td>*16*|**26**</td></tr>
<tr><td>14</td><td>*94.5*|**95.6**</td><td>*88*|**86**</td><td>*1.73*|**1.43**</td><td>*31.9*|**32.9**</td><td>*32.9*|**36.4**</td><td>*8.47*|**5.96**</td><td>*59*|**170**</td><td>*21.7*|**76.1**</td><td>*4*|**27**</td></tr>
<tr><td>15</td><td>*99.0*|**98.6**</td><td>*90*|**84**</td><td>*1.58*|**1.53**</td><td>*31.5*|**32.0**</td><td>*36.2*|**36.0**</td><td>*8.66*|**6.86**</td><td>*56*|**108**</td><td>*25.6*|**58.3**</td><td>*10*|**21**</td></tr>
<tr><td>Avg</td><td>*96.4*|**96.4**</td><td>*89.0*|**85.8**</td><td>*1.56*|**1.45**</td><td>*32.7*|**33.0**</td><td>*34.4*|**34.8**</td><td>*8.93*|**6.3**</td><td>*56*|**151**</td><td>*22.9*|**69.3**</td><td>*11*|**24.8**</td></tr>
<tr><td>Std</td><td>*1.75*|**1.74**</td><td>*1.0*|**1.5**</td><td>*0.14*|**0.11**</td><td>*1.3*|**1.6**</td><td>*2.2*|**1.3**</td><td>*0.73*|**0.43**</td><td>*2*|**26**</td><td>*1.8*|**8.8**</td><td>*5.2*|**2.3**</td></tr>
<tr><td>*p*</td><td>0.45</td><td>0.018</td><td>0.11</td><td>0.58</td><td>0.8</td><td>0.0007</td><td>.001</td><td>0.0004</td><td>0.007</td></tr>
<tr><th>Case #</th><th>Coverage (%)</th><th>Isodose (%)</th><th>nCI</th><th>Chiasm gEUD (Gy)</th><th>Brainstem gEUD (Gy)</th><th>R50%</th><th>Num. of Beams</th><th>Total MU ($10^3$)</th><th>Time (min)</th></tr>
<tr><td>16</td><td>*95.1*|**97.0**</td><td>*93.5*|**92**</td><td>*1.15*|**1.18**</td><td>*53.8*|**51.7**</td><td>*58.6*|**58.6**</td><td>*3.14*|**2.87**</td><td>*50*|**65**</td><td>*10.8*|**16.7**</td><td>*15*|**19**</td></tr>
<tr><td>17</td><td>*98.4*|**96.9**</td><td>*93*|**92**</td><td>*1.29*|**1.24**</td><td>*52.6*|**52**</td><td>*40.7*|**37.3**</td><td>*6.69*|**3.51**</td><td>*35*|**61**</td><td>*13.0*|**15.6**</td><td>*11*|**18**</td></tr>
<tr><td>18</td><td>*96.3*|**95.1**</td><td>*89.7*|**91**</td><td>*1.21*|**1.16**</td><td>*30*|**34**</td><td>*26.2*|**25.4**</td><td>*2.55*|**3.14**</td><td>*50*|**123**</td><td>*13.3*|**21.9**</td><td>*13*|**28**</td></tr>
<tr><td>19</td><td>*96.4*|**94.8**</td><td>*93.7*|**91**</td><td>*1.30*|**1.15**</td><td>*2.2*|**2.3**</td><td>*0.9*|**1.3**</td><td>*3.5*|**2.49**</td><td>*27*|**39**</td><td>*8.0*|**10.2**</td><td>*9*|**15**</td></tr>
<tr><td>20</td><td>*96.3*|**95.1**</td><td>*93.2*|**93**</td><td>*1.14*|**1.09**</td><td>*45.3*|**48.9**</td><td>*52.5*|**52.2**</td><td>*4.67*|**2.84**</td><td>*50*|**33**</td><td>*9.6*|**10.4**</td><td>*9*|**14**</td></tr>
<tr><td>Avg</td><td>*96.5*|**95.8**</td><td>*92.6*|**91.8**</td><td>*1.22*|**1.16**</td><td>*36.8*|**37.8**</td><td>*35.8*|**35.0**</td><td>*4.11*|**2.97**</td><td>*42.4*|**64**</td><td>*11.0*|**15.0**</td><td>*11.5*|**19**</td></tr>
<tr><td>Std</td><td>*1.2*|**1.1**</td><td>*1.65*|**0.8**</td><td>*0.07*|**0.06**</td><td>*21.6*|**21.1**</td><td>*23.1*|**22.9**</td><td>*1.64*|**0.38**</td><td>*10.8*|**36**</td><td>*2.2*|**4.9**</td><td>*2.6*|**5.5**</td></tr>
<tr><td>*p*</td><td>0.33</td><td>0.29</td><td>0.14</td><td>0.46</td><td>0.29</td><td>0.16</td><td>0.21</td><td>0.048</td><td>0.02</td></tr>
</table>

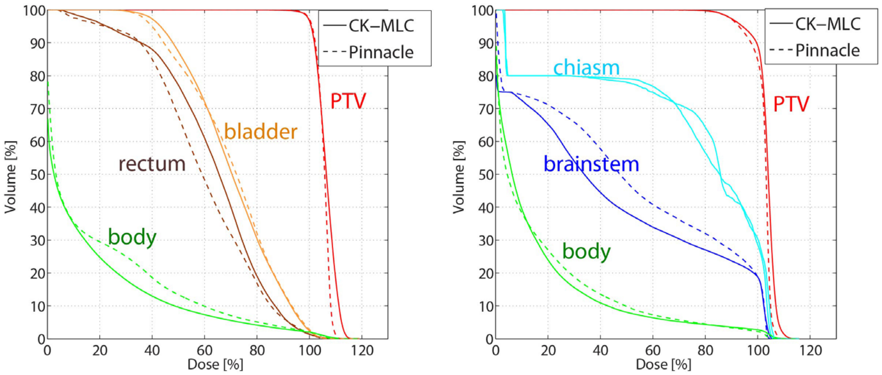

FIG. 5. Average DVHs for the five whole pelvis (left) and five intracranial (right) IMRT plans. The solid lines are for the CK-MLC plans and the dashed lines are for the plans generated for a Siemens linac.

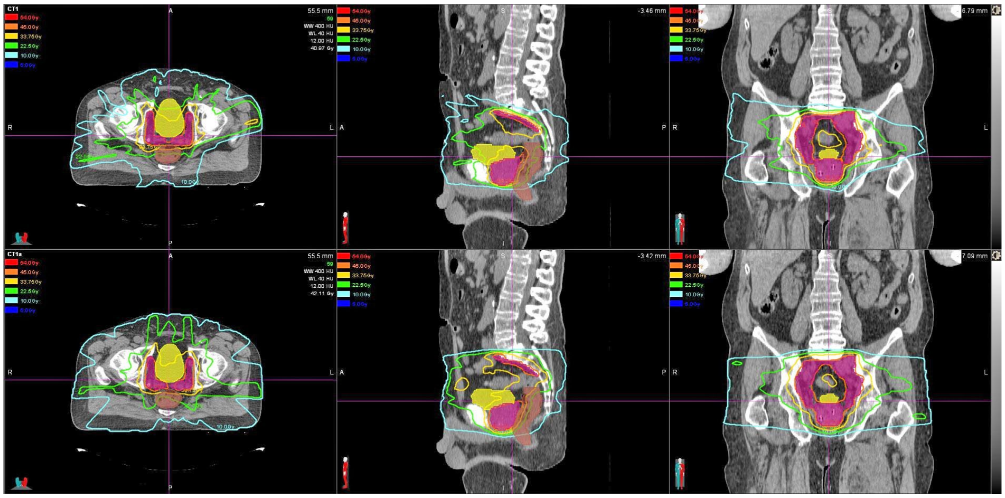

Fig. 6. CT images are shown with isodose lines for Case 11. The top row of images corresponds to the CK-MLC plan and the bottom row of images corresponds to the clinically approved IMRT plan. Isodose lines are shown for 54, 45, 33.75, 22.5, 10, and 5 Gy for a prescription of 45 Gy in 25 fractions.

## IV. DISCUSSION

In this study we generated treatment plans for 10 prostate and 10 intracranial cases using commercially available treatment planning software for the CK-MLC system. We were able to generate hypofractionated SBRT plans that were of equal or better plan quality than clinically approved CyberKnife plans using circular collimators. The total monitor units were reduced by 40% for the prostate cases and 70% for the brain cases, and treatment time was reduced by half. Treatment time slots of 20 to 30 min, including 5 min for setup, would be possible for SRS or SBRT treatments on the CK-MLC, alleviating one of the barriers limiting the wider adoption of CK treatments.

We were able to generate standard fractionated IMRT-like plans with high prescription isodose values (86% on average for the whole pelvis and 92% for the intracranial cases). These prescription isodose values are much higher than is commonly achieved in CK plans using circular collimators and are within 2%–3% of the IMRT plans generated for a conventional linac. Other metrics of plan quality, including conformity and dose to critical organs, were equivalent between both modalities. The large number of noncoplanar beams resulted in lower R50% for the CK plans, at the cost of more MUs and longer treatment times. The larger number of MUs contributes to a higher integral dose to the patient and needs to be carefully evaluated on a case-by-case basis. Even with the longer treatment time, four of the five intracranial IMRT plans generate on CK-MLC had estimated treatment times under 20 min.

The large target volumes for both the whole pelvis (426 $cm^3$ to 760 $cm^3$) and the intracranial (47 $cm^3$ to 177 $cm^3$) cases demonstrate the ability to treat large tumors with the CK-MLC system. However, the plan quality (primarily target dose homogeneity) and treatment efficiency drop when the maximum size of the 2D projection of the target in the beams eye view is larger than the maximum field size of the MLC (10 cm by 12 cm), which was the case for the whole pelvis plans (13.2 cm to 18 cm). These plans required more beams to cover the entire PTV resulting in plans with higher monitor units. The intracranial plans used 64 segments on average (range 33 to 123), while the whole pelvis plans used 151 segments on average (range 110–173). This explains the larger discrepancy in total monitor units and treatment time between the CK-MLC plans and the linac plans for the whole pelvis cases than for the intracranial plans. The impact on treatment time and monitor units for cases with PTV larger than the MLC field size (> 10 cm), but smaller than the whole pelvis plans included here, was not evaluated in this study. It is reasonable to expect the impact to be proportional to the size of the PTV, somewhere between the whole pelvis and intracranial plans evaluated here.

The mechanical accuracy and intrafraction tracking capabilities of the CyberKnife would allow smaller PTV margins and, as a consequence, would necessarily result in lower dose to the surrounding tissue and reduced treatment time than is presented here. For example, Maund et al.[33] find the probability for rectal toxicity greater than grade 2 decreases from 2.1% to 1.4% for a reduction of PTV margin reduction of 5 mm to 3 mm. We chose to use the same PTVs for the CK-MLC plans to focus on the capabilities of the treatment machine and planning system and not on the benefits derived from reduced margins.

## V. CONCLUSIONS

The 10 SBRT plans demonstrate superior performance for the CK-MLC compared to the CyberKnife with circular collimators. A more uniform dose distribution was achieved across the target for equivalent target coverage and conformity. Dose to several important critical organs and to the surrounding tissue was equivalent or reduced for the CK-MLC plans. And finally, the total MUs were reduced by 40% to 70%, and treatment times were reduced by half. The CK-MLC was also able to produce standard fractionated treatment plans prescribed to high isodose lines, while maintaining high conformity, sufficient target coverage, sparing of the adjacent critical organs, and low dose to the surrounding tissue. The total monitor units were up to three times greater for the CK-MLC based plans compared to the linac plans for volume larger than the CK-MLC maximum field size. Treatment times were 19 min, on average, for the standard fractionated intracranial cases planned with the CK-MLC and 25 min, on average, for the whole pelvis cases. The CK-MLC system provides more efficient SRS and SBRT treatments and, in select clinical cases, might be a potential alternative for standard fractionated treatments at centers with only a CK system or as a redundancy to conventional linacs in the event of extended downtime.

## References

1. Furweger C, Drexler C, Kufeld M, Muacevic A, Wowra B. Advances in fiducial-free image-guidance for spinal radiosurgery with CyberKnife — a phantom study. J Appl Clin Med Phys. 2010;12(2):20–28.
2. Fu D and Kuduvalli G. A fast, accurate, and automatic 2D-3D image registration for image-guided cranial radiosurgery. Med Phys. 2008;35(5):2180–94.
3. Murphy MJ. Fiducial-based targeting accuracy for external-beam radiotherapy. Med Phys. 2002;29(3):334–44.
4. Schweikard A, Shiomi H, Adler J. Respiration tracking in radiosurgery. Med Phys. 2004;31(10):2738.
5. Furweger C, Drexler C, Kufeld M, Muacevic A, Wowra B, Schlaefer A. Patient motion and targeting accuracy in robotic spinal radiosurgery: 260 single-fraction fiducial-free cases. Int J Radiat Oncol Biol Phys. 2010;78(3):937–45.
6. Ho AK, Fu D, Cotrutz C, et al. A study of the accuracy of cyberknife spinal radiosurgery using skeletal structure tracking. Neurosurgery. 2007;60(2 Suppl 1):147–56.
7. Kilby W, Dooley JR, Sayeh S, Kuduvalli G, Maurer CR. The CyberKnife robotic radiosurgery system in 2010. Technol Cancer Res Treat. 2010;9(5):433–52.
8. Accuray Inc. CyberKnife M6 series technical specifications. Madison, WI: Accuray; 2013.
9. Xie Y, Djajaputra D, King CR, Hossain S, Ma L, Xing L. Intrafractional motion of the prostate during hypofractionated radiotherapy. Int J Radiat Oncol Biol Phys. 2008;72(1):236–46.
10. Murphy MJ. Intrafraction geometric uncertainties in frameless image-guided radiosurgery. Int J Radiat Oncol Biol Phys. 2009;73(5):1364–68.
11. Echner GG, Kilby W, Lee M, et al. The design, physical properties and clinical utility of an iris collimator for robotic radiosurgery. Phys Med Biol. 2009;54(18):5359–80.
12. Poll JJ, Hoogeman MS, Prevost JB, Nuyttens JJ, Levendag PC, Heijmen BJ. Reducing monitor units for robotic radiosurgery by optimized use of multiple collimators. Med Phys. 2008;35(6):2294–99.
13. Hossain S, Xia P, Huang K, et al. Dose gradient near target-normal structure interface for nonisocentric CyberKnife and isocentric intensity-modulated body radiotherapy for prostate cancer. Int J Radiat Oncol Biol Phys. 2010;78(1):58–63.

14. van de Water S, Hoogeman MS, Breedveld S, Heijmen BJ. Shortening treatment time in robotic radiosurgery using a novel node reduction technique. Med Phys. 2001;38(3):1397–405.
15. Fan J, Hayes S, Li J, Ma C. Multileaf collimator based robotic radiotherapy. Int J Radiat Oncol Biol Phys. 2011;81(2):S857–S858.
16. Fan J, Li J, Price R, et al. MLC-based CyberKnife radiotherapy for prostate cancer [abstract]. Med Phys. 2010;37(6):3217.
17. van de Water S, Hoogeman MS, Breedveld S, Nuyttens JJ, Schaart DR, Heijmen BJ. Variable circular collimator in robotic radiosurgery: a time-efficient alternative to a mini-multileaf collimator? Int J Radiat Oncol Biol Phys. 2011;81(3):863–70.
18. Krayenbuehl J, Davis JB, Ciernik IF. Dynamic intensity-modulated non-coplanar arc radiotherapy (INCA) for head and neck cancer. Radiother Oncol. 2006;81(2):151–57.
19. Accuray Inc. CyberKnife M6 system incise collimator specifications. Madison, WI: Accuray; 2012.
20. Siemans Healthcare. 160 MLC data sheet. Erlangen, Germany: Siemans Healthcare; 2010.
21. Jeleń U, Söhn M, Alber M. A finite size pencil beam for IMRT dose optimization. Phys Med Biol. 2005;50(8):1747–66.
22. Schlaefer A and Schweikard A. Stepwise multi-criteria optimization for robotic radiosurgery. Med Phys. 2008;35(5):2094–103.
23. Dieterich S and Gibbs IC. The CyberKnife in clinical use: current roles, future expectations. Front Radiat Ther Oncol. 2011;43:181–94.
24. Descovich M, Carrara M, Morlino S, et al. Improving plan quality and consistency by standardization of dose constraints in prostate cancer patients treated with CyberKnife. J Appl Clin Med Phys. 2013;14(5):162–72.
25. Nakamura JL, Varhey LJ, Smith V, et al. Dose conformity of gamma knife radiosurgery and risk factors for complications. Int J Radiat Oncol Biol Phys. 2001;51(5):1313–19.
26. Paddick IA. A simple scoring ratio to index the conformity of radiosurgical treatment plans. Technical note. J Neurosurgery. 2000;93(Suppl 3):219–22.
27. van't Riet A, Mak AC, Moerland MA, Elders LH, van der Zee W. A conformation number to quantify the degree of conformality in brachytherapy and external beam irradiation: application to the prostate. Int J Radiat Oncol Biol Phys. 1997;37(3):731–36.
28. Luxton G, Keall PJ, King CR. A new formula for normal tissue complication probability (NTCP) as a function of equivalent uniform dose (EUD). Phys Med Biol. 2008;53(1):23–36.
29. Niemierko A. Reporting and analyzing dose distributions: a concept of equivalent uniform dose. Med Phys. 1997;24(1):103–10.
30. Niemierko A. A generalized concept of equivalent uniform dose (EUD) [abstract]. Med Phys. 1999;26(6):1100.
31. Rancati T, Fiorino C, Gagliardi G, et al. Fitting late rectal bleeding data using different NTCP models: results from an Italian multi-centric study (AIROPROS0101). Radiother Oncol. 2004;73(1):21–32.
32. Eisbruch A, Ten Haken RK, Kim HM, Marsh LH, Ship JA. Dose, volume, and function relationships in parotid salivary glands following conformal and intensity-modulated irradiation of head and neck cancer. Int J Radiat Oncol Biol Phys. 1999;45(3):577–87.
33. Maund IF, Benson RJ, Fairfoul J, Cook J, Huddart R, Poynter A. Image-guided radiotherapy of the prostate using daily CBCT: the feasibility and likely benefit of implementing a margin reduction. Br J Radiol. 2014; 87(1044):20140459.
